# Amyotrophic lateral sclerosis

**DOI:** 10.1186/1750-1172-4-3

**Published:** 2009-02-03

**Authors:** Lokesh C Wijesekera, P Nigel Leigh

**Affiliations:** 1 MRC centre for Neurodegeneration Research, Department of Clinical Neuroscience, Box 41, Institute of Psychiatry, Kings College London, London, SE5 8AF, UK

## Abstract

Amyotrophic lateral sclerosis (ALS) is a neurodegenerative disease characterised by progressive muscular paralysis reflecting degeneration of motor neurones in the primary motor cortex, corticospinal tracts, brainstem and spinal cord. Incidence (average 1.89 per 100,000/year) and prevalence (average 5.2 per100,000) are relatively uniform in Western countries, although foci of higher frequency occur in the Western Pacific. The mean age of onset for sporadic ALS is about 60 years. Overall, there is a slight male prevalence (M:F ratio~1.5:1). Approximately two thirds of patients with typical ALS have a spinal form of the disease (limb onset) and present with symptoms related to focal muscle weakness and wasting, where the symptoms may start either distally or proximally in the upper and lower limbs. Gradually, spasticity may develop in the weakened atrophic limbs, affecting manual dexterity and gait. Patients with bulbar onset ALS usually present with dysarthria and dysphagia for solid or liquids, and limbs symptoms can develop almost simultaneously with bulbar symptoms, and in the vast majority of cases will occur within 1–2 years. Paralysis is progressive and leads to death due to respiratory failure within 2–3 years for bulbar onset cases and 3–5 years for limb onset ALS cases. Most ALS cases are sporadic but 5–10% of cases are familial, and of these 20% have a mutation of the *SOD1 *gene and about 2–5% have mutations of the *TARDBP *(*TDP-43*) gene. Two percent of apparently sporadic patients have *SOD1 *mutations, and *TARDBP *mutations also occur in sporadic cases. The diagnosis is based on clinical history, examination, electromyography, and exclusion of 'ALS-mimics' (*e.g. *cervical spondylotic myelopathies, multifocal motor neuropathy, Kennedy's disease) by appropriate investigations. The pathological hallmarks comprise loss of motor neurones with intraneuronal ubiquitin-immunoreactive inclusions in upper motor neurones and TDP-43 immunoreactive inclusions in degenerating lower motor neurones. Signs of upper motor neurone and lower motor neurone damage not explained by any other disease process are suggestive of ALS. The management of ALS is supportive, palliative, and multidisciplinary. Non-invasive ventilation prolongs survival and improves quality of life. Riluzole is the only drug that has been shown to extend survival.

## Disease names

Amyotrophic lateral sclerosis (ALS), Motor neurone disease (MND), Charcot's disease, Lou Gehrig's disease

## Included diseases

Amyotrophic lateral sclerosis (ALS) is a term used to cover the spectrum of neurodegenerative syndromes characterised by progressive degeneration of motor neurones. However, it is also the term used in modern clinical practice to indicate the commonest form of the disease, Classical (Charcot's) ALS. Other syndromes related to this spectrum of disorders include, Progressive bulbar palsy (PBP), Progressive muscular atrophy (PMA), Primary lateral sclerosis (PLS), Flail arm syndrome (Vulpian-Bernhardt syndrome), Flail leg syndrome (Pseudopolyneuritic form) and ALS with multi-system involvement (*e.g.*, ALS-Dementia). Lord Russell Brain proposed the term Motor neurone disease (MND) to incorporate these conditions into a single spectrum of disorders [1]. The terms 'bulbar onset ALS' and 'spinal onset ALS' have largely replaced the terms PBP and Charcot's ALS in current practice. These syndromes share a common molecular and cellular pathology comprising of motor neurone degeneration and the presence of characteristic ubiquitin-immunoreactive (Ub-IR) and TDP-43 immunoreactive (TDP43-IR) intraneuronal inclusions, as described later [2-4].

Another group of neurodegenerative motor neurone disorders referred to as adult-onset spinal muscular atrophies (*e.g.*, Kennedy's syndrome) which, while affecting anterior horn cells of the spinal cord and/or brainstem, are not considered in this article as they have a distinct molecular pathology unrelated to ALS, and have a more benign disease course.

## Definition and diagnostic/classification criteria

ALS can be defined as a neurodegenerative disorder characterised by progressive muscular paralysis reflecting degeneration of motor neurones in the primary motor cortex, brainstem and spinal cord. "Amyotrophy" refers to the atrophy of muscle fibres, which are denervated as their corresponding anterior horn cells degenerate, leading to weakness of affected muscles and visible fasciculations. "Lateral sclerosis" refers to hardening of the anterior and lateral corticospinal tracts as motor neurons in these areas degenerate and are replaced by gliosis [5].

Despite advances in investigative medicine over the past century, the diagnosis of ALS is based on the presence of very characteristic clinical findings in conjunction with investigations to exclude "ALS-mimic" syndromes (*e.g. *Cervical radiculomyelopathy). The latter conditions lead to diagnostic error in 5–10% of cases [6,7]. The clinical finding of signs suggestive of combined upper motor neurone (UMN) and lower motor neurone (LMN) that cannot be explained by any other disease process (evident on electrophysiological, imaging, cerebrospinal fluid (CSF) or serological studies), together with progression compatible with a neurodegenerative disorder, is suggestive of ALS. Thus, investigation results alone (*e.g.*, evidence of chronic denervation on electromyography (EMG)) are not adequate for achieving a diagnosis, and must be interpreted in light of the patient's history and clinical findings.

The World Federation of Neurology (WFN) Research Group on Motor Neuron Diseases have developed the 1994 'El Escorial' diagnostic criteria [8] and the revised 2000 'Airlie House' criteria [9] to aid in diagnosing and classifying patients for research studies and drug trials. The revised Airlie House criteria are shown in Table 1, and based on these criteria patients can be classified into 'Clinically definite', 'Clinically probable', 'Clinically probable-Laboratory supported' and 'Clinically possible' categories. In the previous 1994 classification, patients with a pure LMN syndrome were classified into the 'Clinically suspected' category, which was removed from the revised criteria. However, it is well recognised that a significant number of patients who either have a pure LMN syndrome or who early in the course of the disease do not have obvious UMN signs, will undoubtedly have ALS (or a variant) but will not fall into these categories in the revised criteria. Therefore, these criteria are probably more useful for research purposes and therapeutic trials, rather than day-to-day clinical practice. A recent rationalisation of the El Escorial Criteria (the Awaji consensus, see below) [10] simplifies the criteria and in our opinion should be adopted.

**Table 1:** Summary of Revised El Escorial Research Diagnostic Criteria for ALS (Brooks et al., 2000)

The diagnosis of ALS requires:	
1 Evidence of LMN degeneration by clinical, electrophysiological or neuropathological examination;	
2 Evidence of UMN degeneration by clinical examination, and	
3 Progressive spread of symptoms or signs within a region or to other regions, as determined by history or examination,	
	
Together with the absence of:	
[1] Electrophysiological and pathological evidence of other disease that might explain the signs of LMN and/or UMN degeneration, and	
	
[2] Neuroimaging evidence of other disease processes that might explain the observed clinical and electrophysiological signs	
Categories of clinical diagnostic certainty on clinical criteria alone	
	
Definite ALS	
• UMN signs and LMN signs in 3 regions Probable ALS	
• UMN signs and LMN signs in 2 regions with at least some UMN signs rostral to LMN signs	
	
Probable ALS – Laboratory supported	
• UMN signs in 1 or more regions and LMN signs defined by EMG in at least 2 regions	
	
Possible ALS	
• UMN signs and LMN signs in 1 region (together), or	
• UMN signs in 2 or more regions	
• UMN and LMN signs in 2 regions with no UMN signs rostral to LMN signs	

UMN signs: clonus, Babinski sign, absent abdominal skin reflexes, hypertonia, loss of dexterity.

LMN signs: atrophy, weakness. If only fasciculation: search with EMG for active denervation.

Regions reflect neuronal pools: bulbar, cervical, thoracic and lumbosacral.

## Epidemiology

The incidence of sporadic amyotrophic lateral sclerosis (SALS) in the 1990's is reported to be between 1.5 and 2.7 per 100,000 population/year (average 1.89 per 100,000/year) in Europe and North America [11], with a uniform incidence across these countries. The point prevalence in the 1990's ranges from 2.7 to 7.4 per 100,000 (average 5.2 per 100,000) in western countries [11]. The lifetime risk of SALS by the age of 70 has been estimated at 1 in 1,000 [12,13] but a more accurate estimate is more likely to be 1 in 400 [14,15]. A consistent finding in studies is that there is a slight excess of males are affected more than females, with a M:F ratio about 1.5:1, although more recent data suggests that the gender ratio may be approaching equality [11,16-18]. Explanations for this male excess have been attributed to possible protective hormonal factors in women, increased likelihood of males being exposed to putative risk factors and under ascertainment of elderly women in some population registers [19,20]. A review published in 2001 found the mortality rates of ALS in the 1990's ranged from 1.54 to 2.55 per 100,000/year and a more recent study estimated the figure to be 1.84 per 100,000 persons in the US population [11,21]. The mean age of onset for sporadic ALS (SALS) varies between 55–65 years with a median age of onset of 64 years[22,23]. Only 5% of cases have an onset before the age of 30 years [23], although juvenile sporadic onset cases are being increasingly recognised [24]. Bulbar onset is commoner in women and in older age groups, with 43% of patients over the age of 70 presenting with bulbar symptoms compared to 15% below the age of 30 [23,25,26].

Although most cases of ALS are sporadic, about 5% of cases have a family history of ALS (Familial ALS; FALS) [27]. There is an often Mendelian inheritance and high penetrance, with most cases having autosomal dominant pattern of inheritance, although autosomal recessive pedigrees have been reported [28,29]. The ages of onset of FALS is about a decade earlier than for sporadic cases, affects males and female equally, and have a shorter survival [28,30,31]. Age of onset in FALS has a normal Gaussian distribution, whereas SALS has an age dependant incidence [32]. Juvenile onset ALS (jALS) is a term used when age of onset is less than 25 years [33]. Most cases are autosomal recessive although dominant inheritance linked to chromosome 9q34 (ALS4, *senataxin*) has been reported [34]. Recessive forms have been mapped to chromosome regions 2q33 (ALS2, *alsin*), and 15q12-21 [35,36].

Geographic loci of the Western Pacific form of ALS, where the prevalence is 50–100 times higher than elsewhere world have been reported, although the cause of these aggregations remains elusive [37]. These populations include the Chamorro people of Guam and Marianas island, the Kii peninsula of Honshu Island, and the Auyu and Jakai people of south west New Guinea, in whom ALS is associated with the Parkinsonism and dementia (ALS-PD complex) [38,39]. More recent studies however have shown a decrease in incidence of both ALS and PDC in these areas over the past 40 years, although the incidence of PDC slightly increased during the eighties and nineties [37,40-42].

## Clinical features

The features of ALS were first clearly described as a clinico-pathological entity by Jean Martin Charcot in 1869 and in subsequent articles in 1874 [43,44]. However, before that Bell (1824), Aran (1850), Duchenne (1851), and Cruveilher (1853) made important observations that contributed to the understanding of the clinical and pathological syndrome [45-50].

Approximately two thirds of patients with typical ALS have a spinal form of the disease (classical 'Charcot ALS'). They present with symptoms related to focal muscle weakness where the symptoms may start either distally or proximally in the upper limbs and lower limbs. Rarely, patients may notice focal muscle wasting before onset of weakness, and some patients may present with a spastic paraparesis. Patients may have noticed fasciculations (noticed as involuntary muscle twitching) or cramps preceding the onset of weakness or wasting for some months (or years), but rarely are these the presenting symptoms. The weakness is usually of insidious onset, and patients may notice that symptoms are exacerbated by cold weather. Although it is usually asymmetrical at onset, the other limbs develop weakness and wasting sooner or later, and most patients go on to develop bulbar symptoms and eventually respiratory symptoms (although not necessarily in that sequence). Gradually, spasticity may develop in the weakened atrophic limbs, affecting manual dexterity and gait. During late stages of the disease patients may develop 'flexor spasms', which are involuntary spasms occurring due to excess activation of the flexor arc in a spastic limb. Occasionally encountered symptoms include new bladder dysfunction (such as urgency of micturition), sensory symptoms, cognitive symptoms and multi-system involvement (*e.g. *dementia, parkinsonism).

Patients with bulbar onset ALS usually present with dysarthria of speech, which may initially only be apparent after ingestion of small amount of alcohol. Rarely, patients may present with dysphagia for solid or liquids before noticing speech disturbances. Limbs symptoms can develop almost simultaneously with bulbar symptoms and in the vast majority of cases will occur within 1–2 years. Almost all patients with bulbar symptoms develop sialorrhoea (excessive drooling) due to difficulty swallowing saliva and mild UMN type bilateral facial weakness which affects the lower part of the face. 'Pseudobulbar' symptoms such as emotional lability and excessive yawning are seen in a significant number of cases.

About 5% of cases with ALS present with respiratory weakness without significant limb or bulbar symptoms [51,52]. These patients present with symptoms of type 2 respiratory failure or nocturnal hypoventilation such as dyspnoea, orthopnoea, disturbed sleep, morning headaches, excessive day time somnolence, anorexia, decreased concentration and irritability or mood changes [53].

The examination early in the course of limb onset disease usually reveals focal muscle atrophy especially involving the muscles of the hands, forearms or shoulders in the upper limbs, and proximal thigh or distal foot muscle in the lower limbs. Fasciculations are usually visible in more than one muscle group. Spasticity is evident in the upper limbs by increased tone and a supinator 'catch', and in the lower limbs with a patellar 'catch' and clonus together with hypertonia. Tendon reflexes are pathologically brisk in a symmetrical manner, including the finger jerks in the upper limbs and positive crossed adductor reflex in the lower limbs. Abnormal spread of tendon reflexes beyond the stimulated muscle group may be evident. The Hoffmann's sign may be positive in the upper limbs and plantar response is often extensor. In patients with bulbar dysfunction, dysarthria may arise from either LMN pathology or pseudobulbar palsy from UMN disorder, leading to slow slurred speech or a nasal quality. On examining the cranial nerves, the jaw jerk may be brisk, especially in bulbar-onset disease. An upper motor neurone type facial weakness affects the lower half of the face causing difficulty with lip seal and blowing cheeks, but often varying degrees of UMN and LMN facial weakness coexist. The gag reflex is preserved and is often brisk while the soft palate may be weak. Patients develop fasciculations and wasting of the tongue, and tongue movements are slowed due to spasticity. The rest of the cranial nerves remain intact, although in late stages of the disease patients may very rarely develop a supranuclear gaze palsy [54,55]. Sensory examination is almost always unremarkable. As disease progresses, patients develop the characteristic picture of the combination of upper motor neurone and lower motor neurone signs coexisting within the same central nervous system region, affecting the bulbar, cervical, thoracic and lumbar territories. Respiratory failure and other pulmonary complications are the usual cause of death in ALS. However, patients who are kept alive by tracheostomy assisted ventilation are found to eventually develop a profound state motor paralysis termed the 'totally locked-in state' (TLS), were there is paralysis of all voluntary muscles and varying degrees of oculomotor impairment [56,57].

## Clinical features of variant disorders

Variants of MND have differing clinical presentations, rate of progression and prognosis. Opinion is divided as to whether these syndromes should be classed as separate entities from ALS, although there is evidence that the may be a common molecular pathology.

The syndrome of progressive muscular atrophy (PMA) accounts for 5–10% of patients with MND, and indicates a pure lower motor neurone syndrome without accompanying upper motor neurone signs [58]. It is almost always of limb onset, but patients may eventually develop swallowing difficulties. It is reported that up to 50% of patients may develop UMN signs and go on to develop typical ALS picture [59].

The "flail arm" and "flail leg" variants are initially localised forms with a predominantly lower motor neuron presentation. In the flail arm variant (which also is known as the Vulpian-Bernhardt syndrome [60] and Brachial amyotrophic diplegia [61]), weakness and wasting predominantly affects the proximal upper limb in a symmetrical pattern, leading to severe wasting around the shoulder girdle and the arms hanging flaccidly either side. Typically, the tendon reflexes in the upper limbs are depressed or absent, but patients may have retained reflexes or focal brisk reflexes especially in the unaffected limb when the disease is asymmetrical at the onset. The lower limbs remain strong for some years but eventually spasticity and wasting develops. Swallowing difficulties and diaphragmatic weakness are usually late features [62,63]. In the flail leg syndrome (also known as the Pseudopolyneuritic form of ALS) [64], weakness and wasting begins in the distal lower limbs affecting both lower limbs in a symmetrical manner. Again the clinical features are of a lower motor neurone syndrome with hypotonia and depressed tendon reflexes. Pyramidal signs are usually absent, although it is not unusual for these patients to have focal brisk reflexes in the unaffected limb when the disease is asymmetrical [65]. The unusual clinical picture together with lack of neurophysiological evidence of denervation in other regions can lead to considerable diagnostic delays. These two variants characteristically show slower progression compared to more typical forms of ALS [61,62,66-68].

Primary lateral sclerosis is a clinically progressive pure upper motor syndrome that cannot be attributed to another disease process. There is ongoing debate as to whether this syndrome is in fact an entirely separate disorder to ALS, but there is evidence from pathological studies that hallmarks of ALS such as ubiquitinated inclusions are present in this condition. Patients present with a pure upper motor neurone syndrome with either absent or minimal lower motor neurone signs. It can be difficult to differentiate PLS from ALS during the early stages as some patients with typical ALS may only manifest UMN signs. For this reason, some authors have suggested that LMN signs must be absent for 3 years from onset to confidently diagnose PLS [69]. However, there may be electrophysiological evidence of LMN involvement in PLS patients despite the absence of clinical LMN signs, and some patients may develop wasting of small muscles of the hands, adding to the diagnostic confusion [70,71], a condition called by some authors as "UMN-dominant ALS" [72,73]. Prognosis for PLS is considerably better than for typical ALS [72].

It is recognised that patients with ALS may exhibit a range of cognitive abnormalities ranging from impaired frontal executive dysfunction in 20–40% of patients, to overt fronto-temporal dementia (FTLD) in approximately 5% of cases [74]. Cognitive abnormalities may precede or occur after the onset of motor symptoms. Neuropathological and neuroimaging studies have indicated that this subset of patients with ALS-dementia may represent a part of spectrum between patients with pure FTLD and ALS [75,76].

## Aetiology

The cause of ALS/MND is unknown although some genetic risk factors have been identified. Recent reviews on the role of environmental risk factors in the causation of ALS have concluded that there is no consistent association between a single environmental factor and risk of developing ALS. Most authors favour a hypothesis of complex genetic-environmental interaction as the causal factor for motor neuron degeneration [77,78].

Putative exogenous risk factors associated with development of ALS investigated in case-control studies have been reviewed, and are summarised in Appendix 1 [19,39]. By applying an evidenced based approach, it was found that only smoking is likely to be associated with developing ALS, while other risk factors were weakly related. More recent case-control studies have estimated the relative risk (RR) of ALS of 0.8–1.67 in smokers compared to non-smokers [79,80], and an odds ratio (OR) of 1.6 independent of age, level of education and occupation [81].

## Pathogenesis of motor neurone degeneration in ALS

The exact molecular pathway causing motor neurone degeneration in ALS is unknown, but as with other neurodegenerative diseases, is likely to be a complex interplay between multiple pathogenic cellular mechanisms which may not be mutually exclusive [77,78]. These include:

### 1. Genetic factors

20% of cases with autosomal dominant FALS and 2% of patients with SALS show mutations in the Copper-Zinc superoxide dismutase (*SOD1*) gene [82]. Mutations in the gene are though to cause disease through a toxic gain of function rather than causing impairment of the antioxidant function of the SOD1 enzyme [77]. Other genes causing familial MND include *alsin *(ALS2) [83,84], *senataxin *(ALS4) [85], Vesicle associated membrane protein (*VAPB*, ALS8) [86], *Angiogenin *[87,88] and a mutation in the p150 subunit of dynactin (DCTN1) [89,90]. Recently, mutations in *TARDBP gene *(encoding the TAR-DNA binding protein TDP-43) located on chromosome 1p36.22 have been linked to familial and sporadic ALS [91-93]. Several other gene mutations have been identified in sporadic cases which may increase susceptibility to ALS, such as mutations in the KSP repeat region in the NEFH gene (encoding neurofilament heavy subunit) [94,95], apolipoprotein E ∑4 genotype (APOE) [96], decreased expression of EAAT2 protein [97,98] and alterations in the Vascular endothelial growth factor (*VEGF*) gene [99] to name a few (See Table 2).

**Table 2:** Familial ALS (fALS) gene mutations and clinical features

**Familial ALS type**	**Locus (gene address)**	**Gene**	**Inheritance**	**Clinical pattern**	**Mutations**	**Causes sporadic disease**
ALS1	21q	*SOD1*	AD	Classical	> 120	yes

ALS2	2q33	*ALSIN*	AR	Young onset, UMN	10	no

ALS3	18q21		AD	Classical	not known	not known

ALS4	9q34	*SETX*	AD	Young onset, slow	3	Probably not

ALS5	15q15	not known	AR	Young onset	not known	Probably not

ALS6	16q21	not known	AD	Classical	not known	not known

ALS7	20ptel-p13	not known	AD	Classical	not known	not known

ALS8	20q13.3	*VAPB*	AD	Varied	1	no

ALS-FTD	9q21-q22	not known	AD	With FTD	not known	not known

ALS-FTD	9p21.3	not known	AD	With FTD	not known	not known

ALS	14q11.2	*Angiogenin*	AD	Classical	6	Yes

FTD (FTD3)	3	*CHMP2B*	AD	FTD (ALS)	2	not known

ALS	1	*TDP43*	AD	ALS	14	Yes

LMND	2p13	*DCTNI*	AD	LMND	1 (+ 4 in ALS?)	Yes?

AD = autosomal dominant; AR = autosomal recessive; CHMP2B = Chromatin modifying protein 2B; DCTNI = dynactin; FTD = frontotemporal lobe dementia; LMND = lower motor neuron disease; SETX = senataxin

VAPB = Vesicle associated membrane protein.

### 2. Excitotoxicity

This is the term for neuronal injury induced by excessive glutamate induced stimulation of the postsynaptic glutamate receptors such as cell surface NMDA receptors and AMPA receptors [77,100]. This over stimulation of glutamate receptors is thought to result in massive calcium influx into the neurons, leading to increased nitric oxide formation and thereby neuronal death. Glutamate levels in CSF are elevated in some patients with ALS [101,102]. This elevation has been attributed to the loss of the glial cell excitatory amino acid transporter EAAT2 [103].

### 3. Oxidative stress

Oxidative stress has longed been linked to neurodegeneration and it is known that accumulation of reactive oxygen species (ROS) cause cell death. As mutations in the anti-oxidant enzyme superoxide dismutase 1 (*SOD1*) gene can cause familial ALS, there is significant interest in this mechanism underlying neurodegenerative process in ALS. This hypothesis is supported by the finding of biochemical changes reflecting free radical damage and abnormal free radical metabolism in CSF and post mortem tissue samples of ALS patients [104-107]. In addition, fibroblasts cultured from ALS patients shows increased sensitivity to oxidative damage controls [108].

### 4. Mitochondrial dysfunction

Abnormalities in mitochondrial morphology and biochemistry have been reported in sporadic ALS patients, SOD1 transgenic mice and cellular models [109-115]. Mitochondria from ALS patients show elevated calcium levels and decreased activity of respiratory chain complexes I and IV, implicating defective energy metabolism [112,116]. Mitochondrial DNA mutations have been described in ALS patients [117-119].

### 5. Impaired axonal transport

Motor neuron axons may reach up to one metre in length in humans, and rely on efficient intracellular transport systems. These systems consist of anterograde (slow and fast) and retrograde transport systems, and rely on molecular 'motors', the kinesin complex of proteins (for anterograde) and the dynein-dynactin complex (for retrograde) [120]. SOD1 transgenic mouse models of ALS show evidence of slowed anterograde transport and retrograde transport [121-124]. Although no such findings have been observed in humans with ALS, mutations in the kinesin genes are known to cause neurodegenerative motor nerve diseases in humans such as hereditary spastic paraplegia and Type 2A Charcot-Marie-Tooth disease [125,126]. Mutations in the dynactin complex cause a lower motor neuron disorder with vocal cord paralysis in humans [89].

### 6. Neurofilament aggregation

Abnormal assembly with accumulation of neurofilaments are commonly seen in several neurodegenerative conditions including SALS and FALS [111,127,128]. In addition, mutations in KSP repeat region of the neurofilament heavy (NFH) gene are found in about 1% of sporadic cases [94,95,129]. Neurofilament proteins together with Peripherin (an intermediate filament protein) are found in the majority of axonal inclusions motor neurones of ALS patients [130]. A toxic isoform of peripherin (peripherin 61), has been found to be toxic to motor neurones even when expressed at modest levels and is detectable in spinal cords of ALS patients but not controls [131].

### 7. Protein aggregation

Intra-cytoplasmic inclusions are a hallmark of both sporadic and familial ALS (See histopathology section). However, it is still unclear as to whether aggregate formation directly causes cellular toxicity and have a key role in pathogenesis, if aggregates may be innocent by-products of the neurodegeneration process, or if formation of the aggregates may actually be a being a beneficial process by being part of a defence mechanism to reduce intracellular concentrations of toxic proteins [77,78].

### 8. Inflammatory dysfunction and contribution of non-neuronal cells

Although ALS is not primarily a disorder of autoimmunity or immune dysregulation, there is considerable evidence that inflammatory processes and non-neuronal cells may play a part in pathogenesis of ALS. Microglial and dendritic cell activation is a prominent pathology in human ALS and transgenic *SOD1 *mice [132-136]. These activated non-neuronal cells produce inflammatory cytokines such as interleukins, COX-2, TNF**α **and MCP-1, and evidence of upregulation is found in CSF or spinal cord specimens of ALS patients or *in vitro *models [137-140]. Despite this evidence, immunomodulatory therapies are yet to show promise as neuroprotective agents in clinical trials of ALS.

### 9. Deficits in neurotrophic factors and dysfunction of signalling pathways

Decreased levels of neurotrophic factors (*e.g. *CTNF, BDNF, GDNF and IGF-1) have been observed in ALS patients post-mortem and in *in vitro *models [141-143]. In addition, deletion of the hypoxia-response element in the vascular endothelial growth factor (*VEGF*) gene was found to cause a motor neurone disease in mice [144]. In humans, three mutations in the *VEGF *gene were found to be associated with increased risk of developing sporadic ALS [99], although a recent meta-analysis by the same authors failed to show an association between VEGF haplotypes and increase the risk of ALS in humans [145].

The final process of cell death in ALS motor neurones is thought to closely resemble a programmed cell death pathway (apoptosis). Biochemical markers of apoptosis are detected in the terminal stages of human and models of ALS [146-150]. Key elements of the normal apoptotic pathway are found to be involved in cell death in ALS, including the caspase family of proteolytic enzymes, the Bcl2 family of oncoproteins (anti-apoptotic and proapoptotic oncogenes) and the apoptosis inhibitor family of proteins (IAPs) [77,151,152].

## Histopathological features

The pathological hallmarks of ALS are the degeneration and loss of motor neurones with astrocytic gliosis and the presence of intraneuronal inclusions in degenerating neurones and glia. Upper motor neurone pathology in ALS is indicated by depopulation of the Betz cells in the motor cortex (Brodmann area 4), variable astrocytic gliosis affecting both the grey matter and underlying subcortical white matter of the motor cortex, and axonal loss within the descending pyramidal motor pathway associated with myelin pallor and gliosis of the corticospinal tracts [153,154].

Lower motor neurone pathology primarily affects the ventral horn motor neurones of the spinal cord and brainstem. There is relative sparing of the motor nucleus of Onufrowicz in the S2 spinal segment and the cranial nerve oculomotor nuclei [4,155]. The number of lower motor neurones can be reduced by up to 50% at autopsy [156] but there is considerable variation both between cases and between different spinal levels within cases [154]. The remaining neurones are atrophic and contain intraneuronal inclusions such as:

### 1. Bunina bodies

These are small eosinophilic, hyaline intracytoplasmic inclusions that stain positive for cystatin and transferring [157,158], and are present in 70–100% of cases [4,159,160]. Also present in Betz cells and subthalamic nuclei. Rarely seen in other conditions.

### 2. Ubiquitinated inclusions or ubiquitin-immunoreactive (UBIs; Ub-IR)

Can be divided according to morphology into skein-like inclusions (SLIs) which have a filamentous profile, and more compact spherical bodies (with a rounded appearance). The compact spherical bodies have also been termed "Lewy-body like" inclusions due to the similarity in their appearance to Lewy bodies found in Parkinson's disease. They are almost universal in ALS and its variants, where it can be seen in up to 95% of cases [2,3,161,162]. It has recently been found that the TAR DNA binding protein 43 (TDP-43) is the major protein constituent in the ubiquitin positive inclusions [163-166].

### 3. Hyaline conglomerate (Neurofilament) inclusions (HCIs)

Associated with FALS and rarely seen in sporadic ALS. These are argyrophilic inclusions seen in spinal cord motor neurones that stain for phosphorylated and non-phosphorylated neurofilaments [167]. They have been also described in other neurodegenerative diseases and normal subjects and are not as specific UBIs [168].

Contrary to early belief that ALS was a disease exclusive to the motor system, there is now significant evidence to suggest that ALS is in fact a multisystem disorder. Extra motor pathology is found in regions such as the frontotemporal cortex, hippocampus, thalamus [169], substantia nigra [170], spinocerebellar pathways [171], dorsal columns [172] and peripheral sensory nerves [173,174].

ALS variant syndromes seem to share a common molecular pathology as suggested by the findings of ubiquitinated inclusions in PLS [175,176], PMA [161], Flail arm syndrome [177,178], Flail leg [179], ALS-dementia [180,181] and Guam ALS-PDC [182]. A recent finding is that the TAR DNA binding protein 43 (TDP-43) has been shown to be a major protein constituent in the ubiquitin positive (tau and **α**-synuclein negative) inclusions found in upper and lower motor neurones in ALS, frontotemporal lobar degeneration with MND (FTLD-MND) and frontotemporal lobar degeneration with ubiquitin inclusions (FTLD-U) [163]. TDP-43 positive inclusions were also detected in one of two cases of PLS but appear to be negative in the inclusions seen in *SOD1 *positive familial ALS [183-185].

## Differential diagnosis

ALS must be differentiated from the "ALS mimic syndromes" which are unrelated disorders that may have a similar presentation and clinical features to ALS or it's variants [5,74]. The most important conditions are shown in Table 3.

**Table 3:** Diagnostic errors and most common 'ALS mimic syndromes'. (Modified from Kato et al., with permission)

**Final diagnosis**	**Characteristic features**	**Distinguishing diagnostic features and investigations**
Cerebral lesions	Focal motor cortex lesions very rarely mimic ALS, but frontal lesions with co-existent cervical or lumbo-sacral root damage may cause confusion.	MRI/CT; no EMG evidence of widespread chronic partial denervation (CPD) in limbs

Skull base lesions	Lower cranial nerve signs (bulbar symptoms and signs; wasting of tongue, often asymmetrical); seldom significant long tract signs unless foramen magnum involved in addition	MRI; CT with bone windows; no EMG evidence of CPD in limbs unless wasting of C8/T1 muscles (rare, but present in some lesions at foramen magnum or high cervical level)

Cervical spondylotic myelopathy	Progressive limb weakness. Asymmetrical onset; combined UMN and LMN signs in arm(s); spastic paraparesis; occasionally fasciculations in arms.	Pain in root distribution, but pain may not be severe and may resolve quickly; often progression followed by clinical stabilisation; no bulbar involvement; MRI evidence of spinal cord and root compression; no evidence of CPD on EMG (NB: patients may have co-existent lumbo-sacral motor radiculopathy with lower limb denervation)

Other cervical myelopathies • Foramen magnum lesions • Intrinsic and extrinsic tumours • Syringomyelia	Progressive weakness; foramen magnum lesions and high cervical cord lesions may be associated with focal (C8/T1) wasting; syringomyelia usually associated with LMN signs and dissociated sensory loss	Usually involvement of cerebellar and/or sensory pathways; MRI of head and cervical spine reveal pathology

Conus lesions and lumbo-sacral radiculopathy	Progressive mixed UMN and LMN syndrome	Usually significant sensory symptoms if not signs; bladder involvement; MRI thoracic and lumbo-sacral region; EMG evidence of radiculopathy

Inclusion body myositis (IBM)	Progressive weakness; bulbar symptoms; sometimes respiratory muscle weakness	Characteristic wasting and weakness of deep finger flexors and quadriceps femoris; EMG evidence of myopathy; muscle biopsy as definitive test (rimmed vacuoles)

Cramp/fasciculation/myokymia syndromes	Cramps, undulating muscle contractions, +/- weakness, stiffness (Isaac's syndrome; peripheral nerve hyper-excitability syndrome)	EMG evidence of myokymia; ~30% VGKC antibodies; ~20% associated with thymoma or lung cancer; association with other autoimmune diseases

Multifocal motor neuropathy (MFMN)	Focal asymmetrical onset, often upper limb; pure LMN syndrome; may stabilise for months or years; M:F 4:1;	Conduction block on nerve conduction studies (NCS); weakness often out of proportion to wasting; improvement with intravenous immunoglobulin (IVIG) in ~70%

Kennedy's disease (X-linked bulbar and spinal muscular atrophy)	Males symptomatic; slowly progressive bulbar and limb weakness	Family history; fasciculations of facial muscles; gynaecomastia; proximal symmetrical weakness in addition to foot drop; mild sensory neuropathy on NCS; positive DNA test for CAG repeat mutation in exon 1 of androgen receptor gene

## Diagnostic methods

### Electrophysiological studies

Patients in whom a diagnosis of ALS is suspected on clinical grounds should have electrophysiological studies primarily to document lower motor dysfunction in clinically involved and uninvolved regions, and secondarily to exclude other disease processes. The first published criteria for electrodiagnosis of ALS were by Lambert in 1957 and 1969 [186,187]. The revised El-Escorial criteria [9] have proposed electrophysiological criteria for the diagnosis of ALS, which have been future refined in December 2006 at an consensus conference on Awaji Island, Japan [10]. It is important to bear in mind that clinical neurophysiological examination is used in the diagnosis of ALS when the diagnosis is clinically suspected, and suggestive neurophysiological abnormalities alone cannot clinch the diagnosis without clinical support.

#### 1. Nerve conduction studies (motor and sensory)

Nerve conduction studies are required for the diagnosis principally to define and exclude other disorders of peripheral nerve, neuromuscular junction and muscle that may mimic or confound the diagnosis of ALS, and these studies should generally be normal or near normal, unless the compound muscle potential is small [9]. In ALS, the distal motor latency (DML) and motor conduction velocity (MCV) remain almost normal, never falling below 70% of the upper or lower limit of normal [188-190]. Motor studies are also important in excluding multifocal motor neuropathy, by the detection of partial conduction block. A marked reduction of proximal amplitude or negative-peak area as compared with the distal ones (over 50%), in short segments, (excluding entrapment sites) implies partial conduction block [191]. F-wave studies are particularly useful in assessing proximal conduction and abnormalities have been reported in ALS. These include increased F-wave latency with normal frequency and increased amplitude, and slowing of F-wave velocity with decreased F-wave frequency. Prominent UMN features may be associated with an increased F-wave frequency [188].

The sensory nerve conduction studies can be abnormal in the presence of entrapment syndromes and coexisting peripheral nerve disease [9]. There is also recent evidence sub-clinical involvement of the sensory system in 10–20% of patients with ALS, suggesting an additional polyneuropathy or sensory ganglionopathy [192,193].

#### 2. Conventional electromyography

Concentric needle electromyography (EMG) provides evidence of LMN dysfunction which is required to support a diagnosis of ALS, and should be found in at least two of the four CNS regions: brainstem (bulbar/cranial motor neurons), cervical, thoracic, or lumbosacral spinal cord (anterior horn motor neurons). For the brainstem region it is sufficient to demonstrate EMG changes in one muscle (*e.g. *tongue, facial muscles, jaw muscles). For the thoracic spinal cord region it is sufficient to demonstrate EMG changes either in the paraspinal muscles at or below the T6 level or in the abdominal muscles. For the cervical and lumbosacral spinal cord regions at least two muscles innervated by different roots and peripheral nerves must show EMG changes [9].

The revised El-Escorial criteria require that both evidence of active or ongoing denervation and chronic partial denervation is required for the diagnosis of ALS, although relative proportions vary from muscle to muscle [9]. Signs of active denervation consist of:

1. fibrillation potentials

2. positive sharp waves

Signs of chronic denervation consist of:

1. large motor unit potentials of increased duration with an increased proportion of polyphasic potentials, often of increased amplitude

2. reduced interference pattern with firing rates higher than 10 Hz (unless there is a significant UMN component, in which case the firing rate may be lower than 10 Hz)

3. unstable motor unit potentials.

Fasciculation potentials are an important characteristic finding in ALS, although they can be seen in normal muscles (benign fasciculations) and are not present in all muscles in ALS patients. In benign fasciculations the morphology of the fasciculation potentials are normal, whereas in fasciculation potentials associated with neurogenic change there are abnormal and complex morphology [10,194]. The Awaji group suggest that the presence of abnormal complex fasciculation potentials in a muscle showing neurogenic change, can be considered equivalent in importance to fibrillation potentials or positive sharp waves [10].

#### 3. Transcranial magnetic stimulation and Central motor conduction studies

Transcranial magnetic stimulation (TMS) allows a non-invasive evaluation of corticospinal motor pathways, and allows detection of UMN lesions in patients who lack UMN signs. Motor amplitude, cortical threshold, central motor conduction time and silent periods can be easily evaluated using this method [195]. Central motor conduction time (CMCT) is often marginally prolonged to muscles of at least one extremity in ALS patients. Electrophysiological features compatible with UMN involvement include [9]:

1. Up to a 30% increase in central motor conduction time determined by cortical magnetic stimulation and

2. Low firing rates of motor unit potentials on maximal effort.

Marked prolongation in the CMCT is seen in FALS patients with *D90A SOD1 *mutations and patients with the flail arm and flail leg variants [196-198].

#### 4. Quantitative electromyography

Motor unit number estimation (MUNE) is a special electrophysiological technique that can provide a quantitative estimate of the number of axons innervating a muscle or group of muscles. MUNE consists of a number of different methods (incremental, multiple point stimulation, spike-triggered averaging, F-wave, and statistical methods), with each having specific advantages and limitations. Despite the lack of a perfect single method for performing MUNE, it may have value in the assessment of progressive motor axon loss in ALS, and may have use as an end-point measure in clinical trials [199].

### Neuroimaging studies

The most important use of neuroimaging is in the diagnosis of ALS to exclude treatable structural lesion that mimics ALS by producing varying degrees of UMN and LMN signs, especially in those with clinically probable or possible ALS. The WFN revised criteria state that imaging studies are not required in cases that have clinically definite disease with bulbar or pseudobulbar onset as it is unlikely that structural lesions can mimic clinically definite disease [9]. Magnetic resonance imaging (MRI) can be used in revealing lesions in the corticospinal tracts in ALS. The most characteristic finding in ALS is hyperintensity of the corticospinal tracts on T2-weighted, proton density weighted and FLAIR-weighted MRI, and is best visualised in the brain and brainstem and to a lesser extent in the spinal cord [200-203]. T2 weighted MRI may also show hypointensity of the primary motor cortex, usually along the posterior bank of the precentral gyrus, although this is an inconsistent and non-specific finding [204].

More advanced neuroimaging modalities such as magnetic resonance spectroscopy, diffusion weighted imaging (DWI)/diffusion tensor imaging (DTI), magnetic resonance voxel-based morphometry and functional imaging techniques (fMRI, PET and SPECT) have a limited role in routine clinical practice but have shown promise in understanding pathophysiology of the disease *in vivo*, identification of potential biomarkers of disease progression and identifying disease changes earlier in the course of the disease facilitating earlier diagnosis [205-210].

### Muscle biopsy and neuropathalogical studies

Biopsy of skeletal muscle or other tissues is not required for diagnosis, unless to rule out a mimic syndrome (e.g. Inclusion body myositis). In addition, muscle biopsy may be used to demonstrate LMN dysfunction in a body region when clinical or electrophysiological findings do not support this. Histological findings that are compatible with the diagnosis of ALS include [9]:

• Scattered hypertrophied muscle fibres.

• No more than a moderate number of target or targetoid fibres.

• Fibre type grouping of no more than mild-to-moderate extent.

• The presence of a small number of necrotic muscle fibres.

### Other laboratory studies

There are few other investigations that may be considered mandatory in the work-up of an ALS patient. Clinical laboratory tests that may be abnormal in otherwise typical case of ALS include [9]:

• Muscle enzymes (serum creatine kinase [unusual above ten times upper limit of normal], ALT, AST, LDH)

• Serum creatinine (related to loss of skeletal muscle mass)

• Hypochloremia, increased bicarbonate (related to advanced respiratory compromise)

• Elevated CSF protein (uncommonly more than 100 mg/dl)

## Management

The management of ALS/MND has considerably changed over the past two decades, with a emphasis on coordinated multidisciplinary care between specialist, community based therapists and palliative care teams. Although the condition is considered incurable, many of the symptoms arising during the course of the disease are treatable, and all efforts should be made to improve quality of life and help maintain the patient's autonomy for as long as possible. Advanced directives on end of life care, respiratory and nutritional management during late stages of life are important issues, and should be discussed with patients and relatives at the earliest opportunity that they are willing to do this. Patients with ALS and their relatives are likely to suffer from depression, feelings of hopelessness and anxiety regarding end-of-life issues following the diagnosis or as the disease progresses [211,212]. Therefore, psychological support in the form of counselling and palliative care should be offered to the patients and relatives early [74,213].

### Symptomatic treatments

Symptomatic treatments aim to improve quality of life of patients and care givers. The main symptoms encountered in ALS and their management are shown in Table 4.

**Table 4:** Symptomatic treatments for ALS (with permission from Radunović et al. 2007)

	**Drugs**	**Other treatments**
Cramps	• Carbamazepine	• Physiotherapy
	• Phenytoin	• Physical exercise
	• Quinine (removed from US market)	• Massage
		• Hydrotherapy

Spasticity	• Baclofen	• Physiotherapy
	• Tizanidine	• Hydrotherapy
	• Dantrolene	• Cryotherapy
	• Botulinum toxin type A	

Excessive watery saliva	• Atropine	• Home suction device
	• Hyoscine hydrobromide	• Dark grape juice
	• Hyoscine butylbromide	• Sugar-free citrus lozenges
	• Hyoscine scopoderm	• Nebulisation
	• Glycopyrronium	• Steam inhalation
	• Amitriptyline	• Injections of botulinum toxin into parotid glands
		• Irradiation of the salivary glands

Persistent saliva and bronchial secretions	• Carbocisteine	• Home suction device
	• Propranolol	• Assisted cough insufflator-exsufflator
	• Metoprolol	• Rehydration (jelly or ice)
		• Pineapple or papaya juice
		• Reduced intake of diary products, alcohol, and caffeine

Excessive or violent yawning	Baclofen	

Laryngospasm	Lorazepam	Reassurance

Pain	• Simple analgesics	Comfort (seating, sleeping, day and night care)
	• Non-steroidal anti-inflammatory drugs	
	• Opioids	

Emotional lability	• Tricyclic antidepressant	
	• Selective serotonin-reuptake inhibitors	
	• Levodopa	
	• Dextrometorphan and quinidine	

Communication difficulties		• Speaking techniques
		• Low-tech augmentative and alternative communication tools
		• Voice amplifiers
		• Light writers
		• Scanning systems operated by switches
		• Brain-computer interfaces

Constipation	• Lactulose	• Hydration
	• Senna	• Increased fibre intake

Depression	• Amitriptyline	• Psychological support, counselling
	• Citalopram	

Insomnia	• Amitriptyline	Comfort, analgesia
	• Zolpidem	

Anxiety	Lorazepam	Psychological support, counselling

Fatigue	Modafinil	

### Ventilatory management

Respiratory insufficiency occurs commonly in patients with ALS and is a major cause of mortality. The presenting symptoms of respiratory muscle weakness include dyspnoea on exertion or talking, orthopneoa, disturbed sleep, excessive daytime somnolence, morning headaches, fatigue, anorexia, depression, poor concentration, vivid nightmares and nocturia. Clinical signs evident on examination include tachypnoea, use of accessory breathing muscles, paradoxical movement of the abdomen, weak cough and rarely papilloedema [74,214].

Measurements of the forced vital capacity (FVC) or relaxed (slow) vital capacity (SVC) are the most widely available measures for detecting respiratory decline. Measurement of the Sniff nasal inspiratory pressure (SNIP) is a good measure of diaphragmatic strength and is probably more accurate than vital capacity, although both measurements underestimate respiratory function in patients with bulbar impairment. The American Academy of Neurology (AAN) ALS Practice Parameter (1999) recommends starting non-invasive ventilation when forced vital capacity declines to 50% of the predicted value [215]. However, patients can develop respiratory failure with a forced vital capacity above 70% of the predicted value, therefore a forced vital capacity of 75% or less is probably more appropriate as a threshold for closer monitoring of respiratory symptoms [216-218]. A SNIP of 32% (~25 cms H2O) or less is highly predictive of respiratory failure [219]. Overnight oximetry can reveal episodes of desaturation consistent with nocturnal hypoventilation. Abnormalities of arterial or venous (ear lobe) blood gases, such as respiratory acidosis are a late but important finding that signifies the need for respiratory support.

Respiratory support is usually provided by non-invasive ventilation (NIV) or invasive ventilation *via *tracheotomy. Bi-level positive pressure devices (BiPAP) are the commonly used form of NIV, whereas continuous positive pressure (CPAP) ventilation is not usually helpful [220]. The timing of initiating NIV treatment varies between counties and centres, but most published international guidelines such as those by the EALSC's work shop group [221] and EFNS task force [222], suggest the criteria proposed by the European ALS/MND Consortium and European Neuromuscular Centre workshop on non-invasive ventilation in MND in May 2002, and are shown in Table 5[74].

**Table 5:** Suggested criteria for non-invasive ventilation (NIV): Provisional European consensus criteria for NIV (European ALS/MND Consortium and European Neuromuscular Centre workshop on non-invasive ventilation in MND, May 2002) [with permission from Leigh et al. 2003]

Symptoms related to respiratory muscle weakness. At least one of	
	
• Dyspnoea	
• Orthopnoea	
• Disturbed sleep (not caused by pain)	
• Morning headache	
• Poor concentration	
• Anorexia	
• Excessive daytime sleepiness (ESS > 9)	
	
AND	Evidence of respiratory muscle weakness (FVC ≤ 80% or SNP ≤ 40 cmH2O)
	
AND	Evidence of EITHER:
	significant nocturnal desaturation on overnight oximetry
	OR
	morning ear lobe blood gas pCO_2 _≥ 6.5 kPa

ESS, Epworth sleepiness scale

NIV is usually initially used for intermittent nocturnal support to alleviate symptoms of nocturnal hypoventilation, although as respiratory function worsens, patients tend to require increasing daytime support and eventually continuous support. Observational studies and a recent randomised controlled trial involving 92 ALS patients show that NIV improves survival and quality of life [223,224]. In patients with severe bulbar impairment, NIV improves sleep-related symptoms, but is unlikely to confer a large survival advantage [223].

### Nutritional management

Dysphagia is a common symptom of ALS and leads to increased risk of aspiration, malnutrition, weight loss and dehydration. Malnutrition and dehydration can also occur inpatients whom have severe upper limb weakness, especially if they live alone, as this leads to difficulties in meal preparation or prolonged meal times. ALS is associated with a hyper metabolic state, therefore patients require increased calorie intake [225,226]. Early management of dysphagia includes dietary advice, modification of food consistency (blending solid, adding thickening agents to liquids) and educating patients on special swallowing techniques (such as supraglottic swallowing and postural changes ('Chin tuck manoeuvre') [74,222].

Most guidelines state that supplementary enteral feeding should be considered when body weight falls by > 10% of the pre-diagnostic or baseline weight [74,222]. The three options available for enteric feeding include percutaneous endoscopic gastrostomy (PEG), percutaneous radiologic gastrostomy (PRG) or radiologically inserted gastrostomy (RIG), and nasogastric tube (NGT) feeding. PEG is the standard procedure for enteral feeding, although the procedure requires mild sedation and therefore has implications in patients with respiratory weakness. To minimise risks, evidence suggests that PEG should be performed before VC falls below 50% of predicted [74,227]. Although it may be possible to insert PEG with NIV assistance, PRG/RIG insertion is a better alternative in these patients [228-230]. NGT is a relatively non-invasive option, but is limited by discomfort and problems associated with long term use such as frequent replacement, and should only be considered in patients who cannot undergo PEG or RIG insertion.

### Disease modifying treatments

Despite many clinical trials and various advances in the understanding of ALS, there has been little success in the search for disease modifying or neuroprotective agents. Riluzole is the only approved drug that has been shown to have a modest effect on prolonging life in ALS patients [231-237]. The mechanism of action of riluzole is not entirely certain but is though to include interference with N-methyl-D-aspartate (NMDA) receptor mediated responses, stabilisation of the inactivated state of voltage-dependent sodium channels, inhibition of glutamate release from pre-synaptic terminals, and increasing of extracellular glutamate uptake [238]. The conclusion of a recent Cochrane Collaboration meta-analysis stated that riluzole at 100 mg probably prolongs median survival by 2–3 months when taken for a 18 month duration (in patients clinically probable or definite El Escorial ALS, with symptoms less than 5 years, FVC > 60% and age < 75 years) [239]. The absolute risk reduction with the 100 mg dose at 12 months was 9%, with the numbers needed-to-treat to delay one death (NNT) after 12 months is 11 [239]. The drug is generally well tolerated with the most common side effects being asthenia, nausea, gastrointestinal upset and abnormal liver function tests, and therefore liver function should be regularly monitored during therapy [240].

Over 100 other neuroprotective agents have been studied in animal models and humans. Some agents that have been evaluated in phase II or III human clinical trials and have shown inconclusive evidence or failed to demonstrate effectiveness for routine clinical practice include:

• Subcutaneous recombinant human insulin-like growth factor (IGF-1, ormyotrophin), neurotrophins including brain derived neurotrophic factor, ciliary neurotrophic factor, glial cell line derived neurotrophic factor and oral xaliproden [241-245]

• Ceftriaxone [246]

• Talampanel (8-methyl-7H-1,3-dioxolo(2,3)benzodiazepine)

• Tamoxifen [247]

• Minocycline [248]

• TCH346 [249]

• Coenzyme Q10 [250]

• Vitamin E [251,252]

• Celecoxib [253]

• Creatine [254,255]

• Copaxone [256]

• ONO 2506 – A randomised placebo-controlled investigate efficacy and safety of ONO-2506PO in the presence of riluzole was negative overall in 2004 but showed a trend towards improved survival in those who started the drug within 14 months of onset.

The use of gene therapy approach to deliver neurotrophic factors directly to neurons by means of genetically engineered adeno-associated viruses (AAV) expressing neurotrophic factor genes has been evaluated in *SOD1 *mouse models with some promising results [257], but human studies are not yet underway. Another approach is the use of autologous stem cell transplantation, but to date there have been no convincing results from human studies [258,259]. The recent discovery of the ability to re-programme human skin fibroblast generate pluripotent stem cells (Induced pluripotent stem cells; iPS) [260] would allow patient and disease specific stem cells to produced, leading to better disease models and eventually better autologous cell replacement therapies.

## Prognosis

Analysis of large patient samples drawn from clinic based populations or population registries consistently show that the overall median survival from onset of symptoms for ALS ranges between 2–3 years for bulbar onset cases and 3–5 years for limb onset ALS cases [12,58,261]. Large clinic cohort studies have shown 3 year and 5 year survival tares to be around 48% and 24% respectively, with approximately 4% surviving longer than 10 years [262,263], whereas 5 year survival reported in population based studies is much lower and ranges from 4–30% [12].

Important prognostic indicators of survival consistently arising from population based studies and clinic cohort studies include clinical phenotype (PMA and Flail arm have better prognosis than typical forms) [264], site of onset (bulbar *vs. *limb onset) [17,261,264-266], age of symptom onset [58,261,264-267], shorter time from symptom onset to diagnosis [266], baseline FVC decline [261,265,268], El Escorial category at presentation [17,267] and riluzole use [265,269].

## Abbreviations

(ALS): Amyotrophic lateral sclerosis; (UMN): upper motor neurone; (LMNs): lower motor neurone; (Ub-IR): ubiquitin-immunoreactive; (TDP43-IR): TDP-43 immunoreactive; (EMG): electromyography; (PMA): progressive muscular atrophy; (PLS): primary lateral sclerosis, (MND): motor neurone disease; (PBP): progressive bulbar palsy; (PMA): progressive muscular atrophy; (CSF): cerebrospinal fluid; (WFN): World Federation of Neurology; (FALS): familial ALS; (SALS): sporadic ALS; (jALS): juvenile onset ALS; (ALS-PD complex): ALS associated with the Parkinsonism and dementia; (TLS): 'totally locked-in state'; (FTLD): fronto-temporal dementia; (RR): relative risk; (OR): odds ratio; (VEGF): vascular endothelial growth factor; (ROS): reactive oxygen species; (*SOD1*): superoxide dismutase 1; (NFH): neurofilament heavy; (IAPs): inhibitor family of proteins; (UBIs): ubiquitinated inclusions; (SLIs): skein-like inclusions; (TDP-43): TAR DNA binding protein 43; (HCIs): hyaline conglomerate inclusions; (FTLD-MND): frontotemporal lobar degeneration with MND; (FTLD-U): frontotemporal lobar degeneration with ubiquitin inclusions; (DML): distal motor latency; (MCV): motor conduction velocity; (TMS): transcranial magnetic stimulation; (CMCT): central motor conduction time; (MUNE): motor unit number estimation; (DWI): diffusion weighted imaging; (DTI): diffusion tensor imaging; (FVC): forced vital capacity; (SVC): slow vital capacity; (SNIP): sniff nasal inspiratory pressure; (AAN): American Academy of Neurology; (NIV): non-invasive ventilation; (BiPAP): bi-level positive pressure devices; (CPAP): continuous positive pressure; (PEG): percutaneous endoscopic gastrostomy; (PRG): percutaneous radiologic gastrostomy; (RIG): radiologically inserted gastrostomy; (MRI): magnetic resonance imaging; (NGT): nasogastric tube; (NMDA): N-methyl-D-aspartate; (AAV): adeno-associated viruses; (iPS): induced pluripotent stem cells.

## Competing interests

In the last 5 years PNL has received support for conducting clinical trials, educational grants, and occasional honoraria from Sanofi-Aventis, Novartis, Exonhit, ONO Pharma, Teva, Trophos, and GlaxoSmithKline. He has served on advisory boards and/or trial steering committees for Sanofi-Aventis, ONOPharma, Teva, Roche, Trophos, and GlaxoSmithKline. He has received research funding from the MRC, Wellcome Trust, UK Department of Health, MND Association, ALS association, and The European Union.

## Appendix 1 – Some exogenous risk factors implicated in sporadic ALS

• Age at menopause (females) [270,271]

• Dietary factors [272]

• Electrical injury [273]

• Family history of non-ALS neurodegenerative disease (Parkinson's or Alzheimer's disease) [274]

• Geographical residence (rural, suburban or urban) [275]

• Gulf war service (Male veterans) [276-278]

• Maternal age [279], Number of births (in females) & Birth order [81,279,280], Loss of child [281]

• Occupation [81,282]

• Physical activity [283,284],

• Playing football professionally [285-287]

• Previous poliomyelitis infection [288]

• Race/ethnicity [289]

• Smoking [79-81,290,291]

• Toxin exposure (agricultural chemicals, lead) [282,292]

• Trauma (*e.g. *Head injury) [274,285,293]

• Years of education [81]
